# Poly[(μ_2_-azido-κ^2^ *N* ^1^:*N* ^1^)[μ_2_-5-(8-quinolyl­oxy­methyl)tetra­zolato-κ^4^ *N* ^1^,*O*,*N* ^5^:*N* ^4^]zinc(II)]

**DOI:** 10.1107/S1600536809016924

**Published:** 2009-05-14

**Authors:** Hong-ling Cai

**Affiliations:** a Ordered Matter Science Research Center, Southeast University, Nanjing 210096, People’s Republic of China

## Abstract

In the title compound, [Zn(C_11_H_8_N_5_O)(N_3_)]_*n*_, the Zn atom is hexa­coordinated by five N atoms and one O atom in a distorted octa­hedral geometry. The chelating 5-(8-quinolyloxymeth­yl)tetra­zolate ligands are approximately planar, with a dihedral angle of 3.6 (2)° between the quinoline and tetra­zole planes. Adjacent Zn atoms are linked by two bridging azide ligands across a centre of inversion, and further coordination by one N atom of an adjacent tetra­zole unit forms two-dimensional frameworks in (100). C—H⋯N inter­actions exist between ligands in neighbouring layers.

## Related literature

For the use of tetra­zole derivatives in coordination chemistry, see: Wang *et al.* (2005 ▶); Xiong *et al.* (2002 ▶). For details of the synthetis, see: Luo & Ye (2008 ▶). For related structures, see: Wang & Ye (2008 ▶); Chen & Ye (2008 ▶).
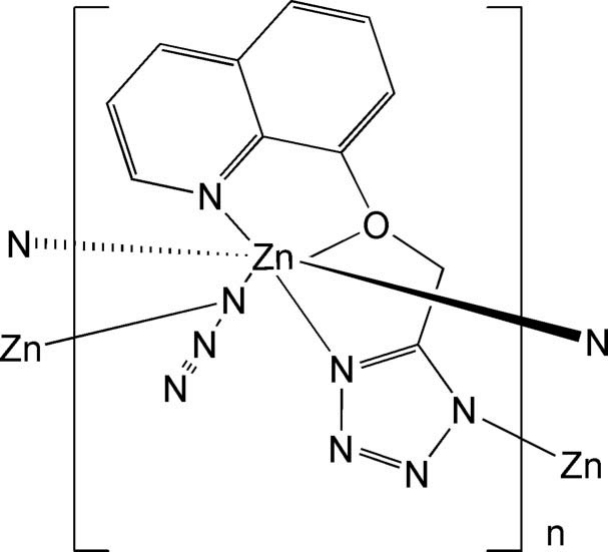


## Experimental

### 

#### Crystal data

[Zn(C_11_H_8_N_5_O)(N_3_)]
*M* *_r_* = 333.64
Monoclinic, 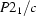

*a* = 10.352 (8) Å
*b* = 14.108 (9) Å
*c* = 8.626 (8) Å
β = 90.31 (2)°
*V* = 1259.8 (17) Å^3^
*Z* = 4
Mo *K*α radiation
μ = 1.96 mm^−1^
*T* = 294 K
0.18 × 0.12 × 0.10 mm

#### Data collection

Rigaku SCXmini CCD diffractometer
Absorption correction: multi-scan *CrystalClear* (Rigaku, 2005 ▶) *T*_min_ = 0.714, *T*_max_ = 0.821
11540 measured reflections
2714 independent reflections
2261 reflections with *I* > 2σ(*I*)
*R*_int_ = 0.059

#### Refinement

*R*[*F* ^2^ > 2σ(*F* ^2^)] = 0.047
*wR*(*F* ^2^) = 0.183
*S* = 1.19
2714 reflections
190 parameters
H-atom parameters constrained
Δρ_max_ = 0.63 e Å^−3^
Δρ_min_ = −0.71 e Å^−3^

### 

Data collection: *CrystalClear* (Rigaku, 2005 ▶); cell refinement: *CrystalClear*; data reduction: *CrystalClear*; program(s) used to solve structure: *SHELXS97* (Sheldrick, 2008 ▶); program(s) used to refine structure: *SHELXL97* (Sheldrick, 2008 ▶); molecular graphics: *SHELXTL* (Sheldrick, 2008 ▶); software used to prepare material for publication: *SHELXTL*.

## Supplementary Material

Crystal structure: contains datablocks I, global. DOI: 10.1107/S1600536809016924/bi2367sup1.cif

Structure factors: contains datablocks I. DOI: 10.1107/S1600536809016924/bi2367Isup2.hkl

Additional supplementary materials:  crystallographic information; 3D view; checkCIF report

## Figures and Tables

**Table 1:** Hydrogen-bond geometry (Å, °)

*D*—H⋯*A*	*D*—H	H⋯*A*	*D*⋯*A*	*D*—H⋯*A*
C3—H3*A*⋯N2^i^	0.93	2.49	3.411 (8)	170
C11—H11*B*⋯N8^ii^	0.97	2.54	3.252 (7)	130

Symmetry codes: (i) 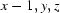; (ii) 
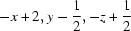
.

**Fig. 1.**
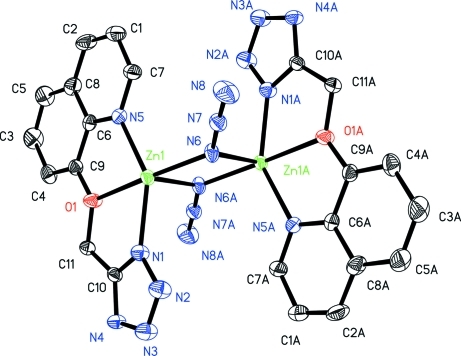
Molecular structure with displacement ellipsoids drawn at the 30% probability level. H atoms are omitted. Symmetry code: (A) 2 - x, 2 - y, -z.

**Fig. 2.**
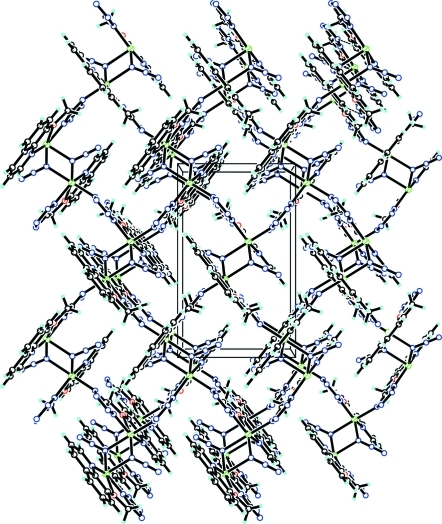
Packing diagram viewed along the a axis.

## supplementary crystallographic information

### Comment

As shown in Fig. 1, the Zn atom adopts a distorted octahedral coordination geometry, coordinated by one N atom and one O atom from 8-hydroxyquinoline, two N atoms from two different tetrazole groups and two N atoms from two bridging azide groups. Thus, 8-[(1*H*-tetrazol-5-yl)methoxy]quinoline acts as a tetradentate linker while the azide groups bridge between Zn atoms to form centrosymmetric rhombic units. Two-dimensional frameworks are formed in the (100) planes, and C—H···N interactions exist between ligands in neighbouring planes.

### Experimental

A mixture of quinolin-8-ol (1.45 g, 10 mmol), 1.38 g K_2_CO_3_, 30 ml acetone and 2-bromoacetonitrile (1.32 g,11 mmol) was refluxed overnight. After cooling, the resulting dark mixture was extracted with ether (30 ml) and the solvent was removed at reduced pressure to give a crude product. Recrystallization from ethanol gave the pure ligand 2-(quinolin-8-yloxy)acetonitrile as a white powder. A mixture of this ligand (0.037 g, 0.2 mmol), ZnCl_2_ (0.026 g, 0.2 mmol) and water (1 ml) was sealed in a glass tube and maintained at 383 K. Yellow crystals of the title compound suitable for X-ray analysis were obtained after 3 d.

### Refinement

H atoms were placed geometrically with C—H = 0.93 or 0.97 Å and allowed to ride during refinement with *U*_iso_(H) = 1.2*U*_eq_(C).

### Crystal data

**Table:**

[Zn(C_11_H_8_N_5_O)(N_3_)]	*F*(000) = 672
*M**_r_* = 333.64	*D*_x_ = 1.759 Mg m^−^^3^
Monoclinic, *P*2_1_/*c*	Mo *K*α radiation, λ = 0.71073 Å
Hall symbol: -P 2ybc	Cell parameters from 3428 reflections
*a* = 10.352 (8) Å	θ = 2.0–27.3°
*b* = 14.108 (9) Å	µ = 1.96 mm^−^^1^
*c* = 8.626 (8) Å	*T* = 294 K
β = 90.31 (2)°	Block, pale yellow
*V* = 1259.8 (17) Å^3^	0.18 × 0.12 × 0.10 mm
*Z* = 4	

### Data collection

**Table:**

Rigaku SCXmini CCD diffractometer	2714 independent reflections
Radiation source: fine-focus sealed tube	2261 reflections with *I* > 2σ(*I*)
graphite	*R*_int_ = 0.059
ω scans	θ_max_ = 27.3°, θ_min_ = 2.0°
Absorption correction: multi-scan *CrystalClear* (Rigaku, 2005)	*h* = −13→13
*T*_min_ = 0.714, *T*_max_ = 0.821	*k* = −18→18
11540 measured reflections	*l* = −11→11

### Refinement

**Table:**

Refinement on *F*^2^	Primary atom site location: structure-invariant direct methods
Least-squares matrix: full	Secondary atom site location: difference Fourier map
*R*[*F*^2^ > 2σ(*F*^2^)] = 0.047	Hydrogen site location: inferred from neighbouring sites
*wR*(*F*^2^) = 0.183	H-atom parameters constrained
*S* = 1.19	*w* = 1/[σ^2^(*F*_o_^2^) + (0.0764*P*)^2^] where *P* = (*F*_o_^2^ + 2*F*_c_^2^)/3
2714 reflections	(Δ/σ)_max_ < 0.001
190 parameters	Δρ_max_ = 0.63 e Å^−^^3^
0 restraints	Δρ_min_ = −0.71 e Å^−^^3^

### Special details

**Table:**

Geometry. All e.s.d.'s (except the e.s.d. in the dihedral angle between two l.s. planes) are estimated using the full covariance matrix. The cell e.s.d.'s are taken into account individually in the estimation of e.s.d.'s in distances, angles and torsion angles; correlations between e.s.d.'s in cell parameters are only used when they are defined by crystal symmetry. An approximate (isotropic) treatment of cell e.s.d.'s is used for estimating e.s.d.'s involving l.s. planes.
Refinement. Refinement of *F*^2^ against ALL reflections. The weighted *R*-factor *wR* and goodness of fit *S* are based on *F*^2^, conventional *R*-factors *R* are based on *F*, with *F* set to zero for negative *F*^2^. The threshold expression of *F*^2^ > σ(*F*^2^) is used only for calculating *R*-factors(gt) *etc*. and is not relevant to the choice of reflections for refinement. *R*-factors based on *F*^2^ are statistically about twice as large as those based on *F*, and *R*- factors based on ALL data will be even larger.

### Fractional atomic coordinates and isotropic or equivalent isotropic displacement parameters (Å^2^)

**Table:**

	*x*	*y*	*z*	*U*_iso_*/*U*_eq_	
Zn1	0.95242 (4)	0.90443 (3)	0.09995 (5)	0.0298 (2)	
N5	0.7775 (3)	0.9622 (3)	0.1958 (4)	0.0347 (8)	
N1	1.0142 (3)	0.7920 (3)	−0.0386 (4)	0.0374 (8)	
O1	0.7733 (3)	0.8144 (2)	0.0151 (4)	0.0420 (8)	
C9	0.6528 (4)	0.8458 (3)	0.0556 (5)	0.0343 (9)	
C8	0.5420 (4)	0.9627 (4)	0.2162 (6)	0.0447 (11)	
C7	0.7806 (5)	1.0369 (3)	0.2914 (5)	0.0433 (11)	
H7A	0.8605	1.0629	0.3173	0.052*	
C6	0.6587 (4)	0.9259 (3)	0.1565 (5)	0.0326 (9)	
C5	0.4240 (5)	0.9206 (4)	0.1716 (8)	0.0595 (15)	
H5A	0.3469	0.9447	0.2100	0.071*	
C4	0.5380 (4)	0.8062 (3)	0.0114 (6)	0.0471 (11)	
H4A	0.5366	0.7553	−0.0571	0.056*	
C3	0.4212 (5)	0.8432 (4)	0.0705 (8)	0.0621 (15)	
H3A	0.3427	0.8161	0.0421	0.075*	
C2	0.5503 (5)	1.0407 (4)	0.3162 (6)	0.0551 (14)	
H2A	0.4755	1.0674	0.3566	0.066*	
C11	0.7891 (4)	0.7426 (3)	−0.0998 (5)	0.0340 (9)	
H11A	0.7493	0.7611	−0.1973	0.041*	
H11B	0.7521	0.6829	−0.0661	0.041*	
N2	1.1345 (4)	0.7624 (3)	−0.0749 (5)	0.0488 (10)	
C10	0.9352 (4)	0.7353 (3)	−0.1149 (5)	0.0319 (8)	
N6	1.0849 (3)	1.0102 (3)	0.1218 (4)	0.0351 (8)	
N3	1.1247 (4)	0.6921 (3)	−0.1722 (5)	0.0504 (11)	
C1	0.6690 (6)	1.0780 (4)	0.3545 (7)	0.0538 (13)	
H1A	0.6753	1.1296	0.4212	0.065*	
N7	1.1303 (4)	1.0413 (3)	0.2394 (4)	0.0382 (8)	
N8	1.1778 (6)	1.0723 (4)	0.3487 (6)	0.0664 (14)	
N4	0.9978 (3)	0.6719 (3)	−0.1977 (4)	0.0371 (8)	

### Atomic displacement parameters (Å^2^)

**Table:**

	*U*^11^	*U*^22^	*U*^33^	*U*^12^	*U*^13^	*U*^23^
Zn1	0.0276 (3)	0.0278 (3)	0.0339 (3)	−0.00349 (16)	0.0000 (2)	−0.00067 (16)
N5	0.0353 (18)	0.0345 (18)	0.0345 (18)	0.0002 (15)	0.0042 (15)	0.0007 (15)
N1	0.0323 (18)	0.039 (2)	0.041 (2)	0.0005 (15)	−0.0040 (16)	−0.0074 (16)
O1	0.0300 (15)	0.0434 (17)	0.0526 (19)	−0.0026 (13)	0.0022 (14)	−0.0189 (14)
C9	0.029 (2)	0.034 (2)	0.040 (2)	−0.0011 (16)	0.0015 (17)	0.0019 (17)
C8	0.032 (2)	0.049 (3)	0.053 (3)	0.011 (2)	0.005 (2)	0.006 (2)
C7	0.046 (3)	0.041 (2)	0.044 (2)	−0.004 (2)	0.003 (2)	−0.011 (2)
C6	0.028 (2)	0.036 (2)	0.034 (2)	−0.0015 (17)	0.0012 (17)	0.0041 (17)
C5	0.030 (3)	0.057 (3)	0.092 (5)	0.008 (2)	0.007 (3)	0.003 (3)
C4	0.036 (2)	0.044 (3)	0.061 (3)	−0.006 (2)	−0.005 (2)	0.004 (2)
C3	0.030 (2)	0.069 (4)	0.087 (4)	−0.005 (2)	−0.005 (3)	−0.001 (3)
C2	0.044 (3)	0.061 (3)	0.061 (3)	0.016 (2)	0.015 (2)	−0.012 (3)
C11	0.038 (2)	0.031 (2)	0.033 (2)	−0.0013 (17)	0.0002 (17)	−0.0049 (16)
N2	0.037 (2)	0.050 (2)	0.059 (3)	0.0069 (18)	−0.0083 (19)	−0.011 (2)
C10	0.039 (2)	0.0271 (19)	0.0290 (18)	−0.0014 (16)	−0.0001 (16)	−0.0019 (15)
N6	0.0348 (18)	0.0364 (19)	0.0341 (18)	−0.0118 (15)	−0.0039 (15)	0.0024 (14)
N3	0.036 (2)	0.054 (3)	0.061 (3)	0.0023 (18)	−0.0036 (19)	−0.019 (2)
C1	0.055 (3)	0.049 (3)	0.057 (3)	0.002 (2)	0.012 (3)	−0.017 (3)
N7	0.041 (2)	0.0360 (19)	0.0371 (19)	−0.0044 (16)	−0.0013 (16)	−0.0009 (15)
N8	0.089 (4)	0.062 (3)	0.048 (3)	−0.012 (3)	−0.018 (3)	−0.013 (2)
N4	0.0325 (18)	0.0384 (19)	0.0403 (19)	0.0055 (15)	−0.0001 (15)	−0.0099 (16)

### Geometric parameters (Å, °)

**Table:**

Zn1—N6	2.035 (4)	C5—C3	1.398 (9)
Zn1—N1	2.088 (4)	C5—H5A	0.930
Zn1—N4^i^	2.102 (4)	C4—C3	1.415 (7)
Zn1—N5	2.155 (4)	C4—H4A	0.930
Zn1—N6^ii^	2.291 (4)	C3—H3A	0.930
Zn1—O1	2.360 (4)	C2—C1	1.376 (8)
N5—C7	1.338 (6)	C2—H2A	0.930
N5—C6	1.373 (6)	C11—C10	1.522 (6)
N1—C10	1.318 (5)	C11—H11A	0.970
N1—N2	1.352 (5)	C11—H11B	0.970
O1—C9	1.371 (5)	N2—N3	1.302 (6)
O1—C11	1.428 (5)	C10—N4	1.318 (5)
C9—C4	1.366 (6)	N6—N7	1.199 (5)
C9—C6	1.426 (6)	N6—Zn1^ii^	2.291 (4)
C8—C2	1.401 (7)	N3—N4	1.361 (6)
C8—C5	1.410 (8)	C1—H1A	0.930
C8—C6	1.415 (6)	N7—N8	1.147 (6)
C7—C1	1.405 (7)	N4—Zn1^iii^	2.102 (4)
C7—H7A	0.930		
			
N6—Zn1—N1	113.67 (17)	C8—C6—C9	118.6 (4)
N6—Zn1—N4^i^	98.71 (15)	C3—C5—C8	120.9 (5)
N1—Zn1—N4^i^	91.07 (17)	C3—C5—H5A	119.5
N6—Zn1—N5	104.72 (17)	C8—C5—H5A	119.5
N1—Zn1—N5	140.15 (14)	C9—C4—C3	119.5 (5)
N4^i^—Zn1—N5	93.41 (15)	C9—C4—H4A	120.2
N6—Zn1—N6^ii^	78.59 (15)	C3—C4—H4A	120.2
N1—Zn1—N6^ii^	88.40 (17)	C5—C3—C4	119.9 (5)
N4^i^—Zn1—N6^ii^	176.76 (13)	C5—C3—H3A	120.1
N5—Zn1—N6^ii^	89.04 (15)	C4—C3—H3A	120.1
N6—Zn1—O1	162.00 (14)	C1—C2—C8	120.0 (5)
N1—Zn1—O1	69.91 (14)	C1—C2—H2A	120.0
N4^i^—Zn1—O1	98.84 (14)	C8—C2—H2A	120.0
N5—Zn1—O1	70.27 (15)	O1—C11—C10	103.0 (3)
N6^ii^—Zn1—O1	83.98 (13)	O1—C11—H11A	111.2
C7—N5—C6	117.7 (4)	C10—C11—H11A	111.2
C7—N5—Zn1	121.1 (3)	O1—C11—H11B	111.2
C6—N5—Zn1	121.1 (3)	C10—C11—H11B	111.2
C10—N1—N2	105.5 (4)	H11A—C11—H11B	109.1
C10—N1—Zn1	123.8 (3)	N3—N2—N1	108.4 (4)
N2—N1—Zn1	130.7 (3)	N4—C10—N1	112.2 (4)
C9—O1—C11	120.9 (3)	N4—C10—C11	125.7 (4)
C9—O1—Zn1	117.5 (3)	N1—C10—C11	122.1 (4)
C11—O1—Zn1	120.3 (2)	N7—N6—Zn1	127.4 (3)
C4—C9—O1	126.0 (4)	N7—N6—Zn1^ii^	125.3 (3)
C4—C9—C6	121.9 (4)	Zn1—N6—Zn1^ii^	101.41 (15)
O1—C9—C6	112.0 (4)	N2—N3—N4	109.7 (4)
C2—C8—C5	123.3 (5)	C2—C1—C7	119.0 (5)
C2—C8—C6	117.6 (5)	C2—C1—H1A	120.5
C5—C8—C6	119.1 (5)	C7—C1—H1A	120.5
N5—C7—C1	123.1 (5)	N8—N7—N6	177.4 (5)
N5—C7—H7A	118.4	C10—N4—N3	104.3 (4)
C1—C7—H7A	118.4	C10—N4—Zn1^iii^	133.6 (3)
N5—C6—C8	122.5 (4)	N3—N4—Zn1^iii^	117.0 (3)
N5—C6—C9	118.8 (4)		
			
N6—Zn1—N5—C7	−19.6 (4)	C5—C8—C6—C9	−1.4 (7)
N1—Zn1—N5—C7	176.1 (3)	C4—C9—C6—N5	−179.8 (4)
N4^i^—Zn1—N5—C7	80.3 (4)	O1—C9—C6—N5	1.8 (6)
N6^ii^—Zn1—N5—C7	−97.6 (4)	C4—C9—C6—C8	2.3 (7)
O1—Zn1—N5—C7	178.5 (4)	O1—C9—C6—C8	−176.1 (4)
N6—Zn1—N5—C6	158.0 (3)	C2—C8—C5—C3	179.5 (5)
N1—Zn1—N5—C6	−6.3 (4)	C6—C8—C5—C3	0.4 (9)
N4^i^—Zn1—N5—C6	−102.1 (3)	O1—C9—C4—C3	176.1 (5)
N6^ii^—Zn1—N5—C6	80.0 (3)	C6—C9—C4—C3	−2.0 (7)
O1—Zn1—N5—C6	−3.9 (3)	C8—C5—C3—C4	−0.1 (10)
N6—Zn1—N1—C10	−154.1 (3)	C9—C4—C3—C5	0.9 (9)
N4^i^—Zn1—N1—C10	106.0 (4)	C5—C8—C2—C1	−180.0 (6)
N5—Zn1—N1—C10	9.3 (5)	C6—C8—C2—C1	−0.8 (8)
N6^ii^—Zn1—N1—C10	−77.3 (4)	C9—O1—C11—C10	176.3 (4)
O1—Zn1—N1—C10	6.9 (3)	Zn1—O1—C11—C10	9.5 (4)
N6—Zn1—N1—N2	23.8 (5)	C10—N1—N2—N3	1.5 (5)
N4^i^—Zn1—N1—N2	−76.2 (4)	Zn1—N1—N2—N3	−176.6 (3)
N5—Zn1—N1—N2	−172.8 (3)	N2—N1—C10—N4	−0.3 (5)
N6^ii^—Zn1—N1—N2	100.6 (4)	Zn1—N1—C10—N4	178.0 (3)
O1—Zn1—N1—N2	−175.2 (4)	N2—N1—C10—C11	177.4 (4)
N6—Zn1—O1—C9	−71.8 (5)	Zn1—N1—C10—C11	−4.3 (6)
N1—Zn1—O1—C9	−176.7 (3)	O1—C11—C10—N4	173.4 (4)
N4^i^—Zn1—O1—C9	95.4 (3)	O1—C11—C10—N1	−3.9 (5)
N5—Zn1—O1—C9	4.9 (3)	N1—Zn1—N6—N7	−123.2 (4)
N6^ii^—Zn1—O1—C9	−86.2 (3)	N4^i^—Zn1—N6—N7	−28.2 (4)
N6—Zn1—O1—C11	95.5 (5)	N5—Zn1—N6—N7	67.7 (4)
N1—Zn1—O1—C11	−9.5 (3)	N6^ii^—Zn1—N6—N7	153.7 (5)
N4^i^—Zn1—O1—C11	−97.4 (3)	O1—Zn1—N6—N7	139.0 (4)
N5—Zn1—O1—C11	172.1 (3)	N1—Zn1—N6—Zn1^ii^	83.13 (19)
N6^ii^—Zn1—O1—C11	81.0 (3)	N4^i^—Zn1—N6—Zn1^ii^	178.17 (15)
C11—O1—C9—C4	9.5 (7)	N5—Zn1—N6—Zn1^ii^	−85.94 (17)
Zn1—O1—C9—C4	176.6 (4)	N6^ii^—Zn1—N6—Zn1^ii^	0.0
C11—O1—C9—C6	−172.2 (4)	O1—Zn1—N6—Zn1^ii^	−14.7 (5)
Zn1—O1—C9—C6	−5.1 (5)	N1—N2—N3—N4	−2.1 (6)
C6—N5—C7—C1	1.2 (7)	C8—C2—C1—C7	0.3 (9)
Zn1—N5—C7—C1	178.9 (4)	N5—C7—C1—C2	−0.5 (9)
C7—N5—C6—C8	−1.8 (6)	N1—C10—N4—N3	−1.0 (5)
Zn1—N5—C6—C8	−179.4 (3)	C11—C10—N4—N3	−178.5 (4)
C7—N5—C6—C9	−179.6 (4)	N1—C10—N4—Zn1^iii^	−153.6 (3)
Zn1—N5—C6—C9	2.8 (5)	C11—C10—N4—Zn1^iii^	28.8 (7)
C2—C8—C6—N5	1.6 (7)	N2—N3—N4—C10	1.9 (5)
C5—C8—C6—N5	−179.2 (5)	N2—N3—N4—Zn1^iii^	160.0 (3)
C2—C8—C6—C9	179.4 (4)		

Symmetry codes: (i) *x*, −*y*+3/2, *z*+1/2; (ii) −*x*+2, −*y*+2, −*z*; (iii) *x*, −*y*+3/2, *z*−1/2.

### Hydrogen-bond geometry (Å, °)

**Table:**

*D*—H···*A*	*D*—H	H···*A*	*D*···*A*	*D*—H···*A*
C3—H3A···N2^iv^	0.93	2.49	3.411 (8)	170
C11—H11B···N8^v^	0.97	2.54	3.252 (7)	130

Symmetry codes: (iv) *x*−1, *y*, *z*; (v) −*x*+2, *y*−1/2, −*z*+1/2.
